# X-ray crystallographic structure of a bacterial polysialyltransferase provides insight into the biosynthesis of capsular polysialic acid

**DOI:** 10.1038/s41598-017-05627-z

**Published:** 2017-07-19

**Authors:** Christian Lizak, Liam J. Worrall, Lars Baumann, Moritz M. Pfleiderer, Gesa Volkers, Tianjun Sun, Lyann Sim, Warren Wakarchuk, Stephen G. Withers, Natalie C. J. Strynadka

**Affiliations:** 1 Department of Biochemistry and Molecular Biology, University of British Columbia, Vancouver, BC V6T 1Z3 Canada; 2 Centre for Blood Research, University of British Columbia, 2350 Health Sciences Mall, Vancouver, BC V6T 1Z3 Canada; 3 Department of Chemistry, University of British Columbia, Vancouver, BC V6T 1Z1 Canada; 4 Department of Biochemistry and Molecular Biology, Centre for High-Throughput Biology, University of British Columbia, Vancouver, BC V6T 1Z4 Canada; 5 Department of Chemistry and Biology, Ryerson University, Toronto, ON M5B 2K3 Canada; 6 LimmaTech Biologics AG, Grabenstrasse 3, 8952 Schlieren, Switzerland

## Abstract

Polysialic acid (polySia) is a homopolymeric saccharide that is associated with some neuroinvasive pathogens and is found on selective cell types in their eukaryotic host. The presence of a polySia capsule on these bacterial pathogens helps with resistance to phagocytosis, cationic microbial peptides and bactericidal antibody production. The biosynthesis of bacterial polySia is catalysed by a single polysialyltransferase (PST) transferring sialic acid from a nucleotide-activated donor to a lipid-linked acceptor oligosaccharide. Here we present the X-ray structure of the bacterial PST from *Mannheimia haemolytica* serotype A2, thereby defining the architecture of this class of enzymes representing the GT38 family. The structure reveals a prominent electropositive groove between the two Rossmann-like domains forming the GT-B fold that is suitable for binding of polySia chain products. Complex structures of PST with a sugar donor analogue and an acceptor mimetic combined with kinetic studies of PST active site mutants provide insight into the principles of substrate binding and catalysis. Our results are the basis for a molecular understanding of polySia biosynthesis in bacteria and might assist the production of polysialylated therapeutic reagents and the development of novel antibiotics.

## Introduction

Sialic acid (Sia) is a negatively charged monosaccharide that is widely found on eukaryotic cell surface glycoconjugates. These nine carbon sugar residues (N-acetylneuraminic acid being the predominant form) play essential roles in a multitude of biological processes such as cell-cell interaction, bacterial infection, and when inappropriately expressed, several forms of cancer^1^. Sia can be found on a handful of eukaryotic proteins as α-2,8-linked homo-polymeric structures of up to 400 residues called polysialic acid (polySia)^2^. Due to its exceptional physicochemical properties, polysialylation strongly promotes cell migration, neuronal plasticity, as well as tumour metastasis. In addition, altered levels of polySia have implications in development, schizophrenia and nerve repair^2–7^. In vertebrates, polysialylation is catalysed by two Golgi-residing polysialyltransferases (PSTs) of the ST8Sia family transferring Sia from the CMP-Neu5Ac donor substrate to the non-reducing termini of sialylated N- and O-linked glycans on glycoproteins^8–10^. The major acceptors of this highly regulated and protein-specific posttranslational modification are neural cell adhesion molecule (NCAM) and synaptic cell adhesion molecule (SynCAM 1) in the nervous system, as well as neuropilin 2 on dendritic cells^11^.

**Figure 1:**
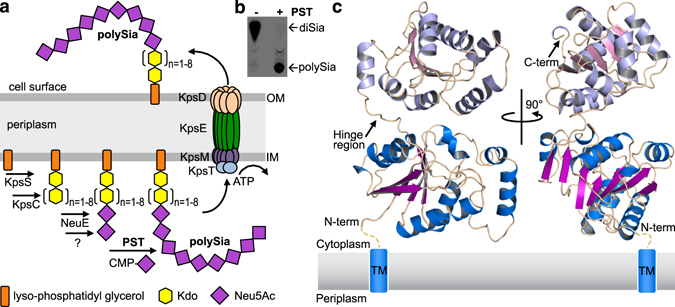
PST catalysed biosynthesis of polysialic acid and structure of *Mh*PST. (**a**) The biosynthesis of capsular polysialic acid (polySia) is initiated at the cytoplasmic side of the plasma membrane by the transfer of a Kdo linker onto the lipid carrier lyso-phosphatidyl glycerol catalysed by KpsS and KpsC. Extension to polySia requires sialic acid priming involving the action of NeuE, before PST can catalyse the assembly of homopolymeric, α-2,8-linked polySia. The completed, lipid-bound structure is translocated to the cell surface in an ATP-dependent manner by an export complex composed of KpsDEMT. (**b**) *In vitro* polysialylation activity of *Mh*PST using a soluble BDP-Sia_2_Lac acceptor substrate was analysed by TLC. Polysialylation results in suppressed migration of the acceptor (A complete scan of the TLC plate is shown in Supplementary Figure S7). (**c**) Ribbon diagrams of the Δ20*Mh*PST apo structure showing the GT-B fold. In the N-terminal Rossmann fold, helices are coloured in marine and β-sheets are coloured in purple, whereas in the C-terminal Rossmann fold, helices are coloured in light blue and β-sheets are coloured in pink. The presumed position of the N-terminal membrane anchor is indicated.

Apart from their specialized presence in vertebrates, polySia modifications also exist in prokaryotes. So far four genera of neuroinvasive Gram-negative bacteria including *Neisseria meningitidis* have been identified to synthesize capsular polysaccharides consisting of polySia that resemble the structures found on eukaryotic glycoproteins. The molecular mimicry of these bacterial polySia capsules represents an elegant strategy to evade the host’s immune recognition since they are not considered as foreign^12, 13^. In addition, they confer a physical barrier protecting the pathogen from killing by the complement system^14^. Bacterial polySia capsules exist in three different flavours: *Escherichia coli* K1, *N. meningitidis* serotype B, *Moraxella nonliquefaciens*, and *Mannheimia haemolytica A2* synthesize α-2,8-linked polySia^15–18^, whereas *N. meningitidis* serotype C produces a α-2,9-linked polymer and *E. coli* K92 produces polymers with alternating α-2,8 and α-2,9 linkages^19–21^. Unlike those in vertebrates, bacterial polySia structures are covalently linked to the lipid carrier lyso-phosphatidyl glycerol^22^ and their biosynthesis follows a general concept conserved for all type 2 capsular polysaccharides. The assembly is initiated at the cytoplasmic side of the plasma membrane by the formation of a β-Kdo linker composed of two to nine Kdo monomers, catalysed by the enzymes KpsS and KpsC (*E. coli* nomenclature)^23, 24^. Sialic acid priming of the glycan has been proposed to involve the putative sialyltransferase NeuE, but several *in vitro* studies have suggested the existence of an additional enzyme to synthesize a di-sialylated structure required for initiation of polysialylation^25–27^. In the central step of the biosynthesis, the formation of the linear α-2,8-linked homo-polymer is catalysed by PST (NeuS) utilizing the nucleotide activated donor substrate CMP-Neu5Ac^28, 29^. The completed, lipid-linked polysaccharide is translocated to the cell surface by the trans-envelope complex KpsDEMT containing the ABC-transporter KpsMT as the driving force^30–32^ (Fig. 1a).

Despite the biochemical characterization of bacterial PSTs^26, 33, 34^, the reaction mechanism of this fundamental enzyme is currently insufficiently understood, mainly due to the lack of structural information. Recent structures of the human ST8SiaIII enzyme provided important insights into polysialylation in mammals^35^, but bacterial PSTs belong to a completely distinct family of glycosyltransferases (Carbohydrate Active Enzyme (CAZy) designated family GT38 distinct from GT29 for ST8SiaIII)^36^, which is not specific for particular acceptor proteins, but assembles polySia on a lipid-linked oligosaccharide^10, 37^.

To understand the unique structural features and the reaction mechanism of bacterial PSTs at a molecular level, we have performed a crystallographic and biochemical characterization of the PST enzyme from *M. haemolytica* serotype A2. We have determined the X-ray structure of the apo enzyme, as well as the structures of complexes with CDP and the pentasaccharide heparin-mimetic fondaparinux, respectively. In combination with detailed kinetic analyses of active site mutants, this work provides essential insights into the structural architecture, as well as into the molecular principles of substrate binding and catalysis of polysialylation in bacteria.

## Results

### Structure of *M. haemolytica* PST

Bacterial PSTs are membrane-associated enzymes acting at the cytoplasmic side of the inner membrane. Since it has been shown that N-terminal truncation of *M. haemolytica* PST (*Mh*PST) lacking the putative membrane anchor segment results in soluble enzyme^34^, we expressed the N-terminal truncation Δ20*Mh*PST within the cytoplasm of *E. coli*. The purified enzyme was able to synthesize a polysialylated product from CMP–Neu5Ac donor and BODIPY-di-sialyllactose (BDP-Sia_2_Lac) acceptor (Fig. 1b), and our kinetic analysis revealed a K_m_ of 0.6 mM for CMP–Neu5Ac (Table 1) consistent with previous reports^33, 34, 38^.

**Table 1:** Kinetic parameters for donor substrate CMP-Neu5Ac.

Mutant	K_*m*_ (mM)	k_*cat*_ (s^−1^)	k_*cat*_/K_*m*_ (s^−1^ M^−1^)
wt	0.60 ± 0.03	70.26 ± 9.62	117,488
Q41A	0.96 ± 0.13	2.23 ± 0.12	2,317
Q44A	0.34 ± 0.03	1.16 ± 0.06	3,397
E152A	1.08 ± 0.05	0.39 ± 0.01	359
E153A	—	—	0.01
R259A	2.08 ± 0.27	0.12 ± 0.03	56
H291A	4.36 ± 0.24	1.83 ± 0.19	420
K293A	0.26 ± 0.04	0.14 ± 0.04	515

Determined at constant acceptor concentration of 0.3 mg/mL colominic acid. Kinetic values are the mean ± s.e.m.

To obtain suitably ordered crystals, we introduced two surface entropy reduction mutations (K68A, K69A). We crystallized the resulting Δ20*Mh*PST construct using the microbatch method and solved the X-ray structure of the apo-enzyme to 2.8 Å resolution (Table 2). Co-crystallization of Δ20*Mh*PST with the acceptor substrate analogue di-sialyl-N-acetyllactosamine-6-sulfate (Sia_2_LacNAc6S) resulted in better-ordered crystals diffracting to 2.2 Å (Table 2), but no clear density for the ligand was observed. This might be a direct consequence of the weak binding of di-sialylated acceptor substrates, as we determined a K_m_ of 2.26 mM for the Sia_2_Lac acceptor (Table 3). A comparison of the apo-structure with the structure determined in the presence of Sia_2_LacNAc6S revealed no significant difference in the overall conformation or in the orientation of individual side chains (r.m.s.d. = 0.27 Å over 5695 atoms). However, the Sia_2_LacNAc6S structure showed unambiguous electron density for two regions that were poorly resolved in the apo-structure (residues M20 to K32 and E231 to K251, neither involved in substrate binding or catalysis, see below). Therefore, we have used this latter more complete structure in our current analysis.

**Table 2:** Data collection and refinement statistics.

Structure	Δ20*Mh*PST apo	Δ20*Mh*PST + CDP	Δ20*Mh*PST + Sia_2_LacNAc6S	Δ20*Mh*PST + fondaparinux
**Data collection**				
Space group	P 31 2 1	P 31 2 1	P 31 2 1	P 31 2 1
Cell dimensions				
a, b, c (Å)	78.08 78.08 303.29	78.28 78.28 303.58	78.26 78.26 301.99	78.31 78.31 299.89
α, β, γ (°)	90 90 120	90 90 120	90 90 120	90 90 120
Resolution (Å)	61.76–2.75 (2.85–2.75)^a^	40.55–3.0 (3.18–3.0)	40.41–2.2 (2.26–2.2)	44.93–3.1 (3.31–3.1)
*CC1/2*	0.999 (0.63)	0.996 (0.576)	0.999 (0.559)	0.998 (0.586)
*R*_*measure*_ (%)	16.9 (193.5)	15.5 (117.4)	5.6 (91.5)	14.7 (149.5)
*Rpim (%)*	5.2 (58.9)	6.7 (49.5)	2.4 (49.1)	5.4 (54.5)
*I/σI*	21.4 (1.9)	8.8 (1.5)	22.0 (1.6)	14.7 (1.5)
Completeness (%)	100 (100)	99.9 (100)	99.9 (99.3)	100 (99.1)
Redundancy	21.6 (22.2)	5.2 (5.5)	5.2 (3.3)	7.2 (7.4)
**Refinement**				
Reflections used in refinement	28972 (2828)	22547 (2183)	55677 (5420)	20263 (1943)
R_*work*_ */*R_*free*_	0.1901/0.2415	0.1942/0.2569	0.1935/0.2279	0.1878/0.2383
**No. atoms**				
Protein	6299	6425	6339	6421
Water	51	32	251	1
Ligand		60		101
**B-factors (Å** ^**2**^ **)**				
Protein	58.9	77.4	48.9	92.8
Water	52.7	58.2		95.9
Ligand		107	51.8	62
**R.M.S deviations**				
Bond lengths (Å)	0.010	0.011	0.017	0.012
Bond angles (°)	1.21	1.62	1.88	1.62
**Ramachandran**				
Favoured (%)	97.23	96.15	96.64	95.07
Allowed (%)	2.5	3.85	3.09	4.66
Outliers (%)	0.26	0.0	0.27	0.27

^a^Numbers in parenthesis refer to the highest resolution shell.

**Table 3:** Kinetic parameters for acceptor substrate Sia_2_Lac.

Mutant	K_*m*_ (mM)	k_*cat*_ (s^−1^)	k_*cat*_/K_*m*_ (s^−1^ M^−1^)
wt	2.26 ± 0.34	69.86 ± 0.59	30,977
wt^a^	1.86 ± 0.06	134.23 ± 0.32	72,103
Q41A	1.02 ± 0.05	3.15 ± 0.00	3,092
Q44A	—	—	0.2
E152A	1.36 ± 0.22	0.38 ± 0.00	280
E153A	—	—	—
R259A	2.57 ± 0.34	0.46 ± 0.00	177
H291A	3.20 ± 0.12	0.76 ± 0.00	239
K293A	—	—	1

Determined at constant donor concentration of 5 mM CMP-Neu5Ac. ^a^Kinetic parameters for acceptor substrate Sia_3_Lac. Kinetic values are the mean ± s.e.m.

We observed a non-crystallographic dimer of *Mh*PST in the asymmetric unit of all determined crystal structures, where the N-terminal loops intertwine with the opposite monomer providing substantial crystal contacts (Supplementary Fig. S1a). In solution *Mh*PST is monomeric as suggested by size exclusion chromatography (data not shown). Superimposition of the two monomers reveals a slight difference in relative orientation between the N- and C-terminal domains caused by structural flexibility in a hinge region connecting the two domains (r.m.s.d. = 1.91 Å over 3052 atoms). However, the individual N- and C-terminal domains superimpose well with r.m.s.d. values of 0.20 Å and 0.17 Å over 1378 atoms and 1085 atoms, respectively (Supplementary Fig. S1b). The *Mh*PST monomer is composed of two non-identical Rossmann-like α/β/α domains structurally separated by the described hinge region (F227 to N236) (Fig. 1c, Supplementary Fig. S1b). The core of the N-terminal domain is formed by seven parallel β-sheets that are flanked by four α-helices on one side and five α-helices on the other side. The slightly shorter C-terminal Rossmann domain is made up of a six-stranded parallel β-sheet surrounded by three and five α-helices, respectively and contains the nucleotide-binding site (see below) (Supplementary Fig. S2). The observed architecture of *Mh*PST reflects a GT-B fold commonly found for metal independent glycosyltransferases^39^. The N-terminal Rossmann domain of *Mh*PST is preceded by a 12 amino acid long tail of extended conformation (Supplementary Fig. S1b), which connects the enzyme to the putative membrane anchor (absent in our truncated Δ20*Mh*PST construct), thereby providing sufficient distance to the plasma membrane (Fig. 1c).

A DALI search with the monomeric *Mh*PST structure finds only proteins with low structural similarity (r.m.s.d. values greater than 3.7 Å), including various GTs and non-GT enzymes (e.g. UDP-GlcNAc 2-epimerases)^40^. Unlike most of these structures, *Mh*PST lacks the GT-B typical C-terminal extension that interacts with the N-terminal domain. Searching the PDB with the individual Rossmann-like domains, resulted in slightly closer matches (r.m.s.d. values around 3.0 Å) and identified, amongst others, bacterial mono-sialyltransferases of the GT80 family, particularly for the search with the C-terminal domain.

Notably *Mh*PST shows no structural similarity to mammalian PSTs of the GT29 family, as the structure of the human ST8SiaIII enzyme exhibits a GT-A fold consisting of a single Rossmann-like domain^35^.

### Nucleotide activated sugar donor binding site

**Figure 2:**
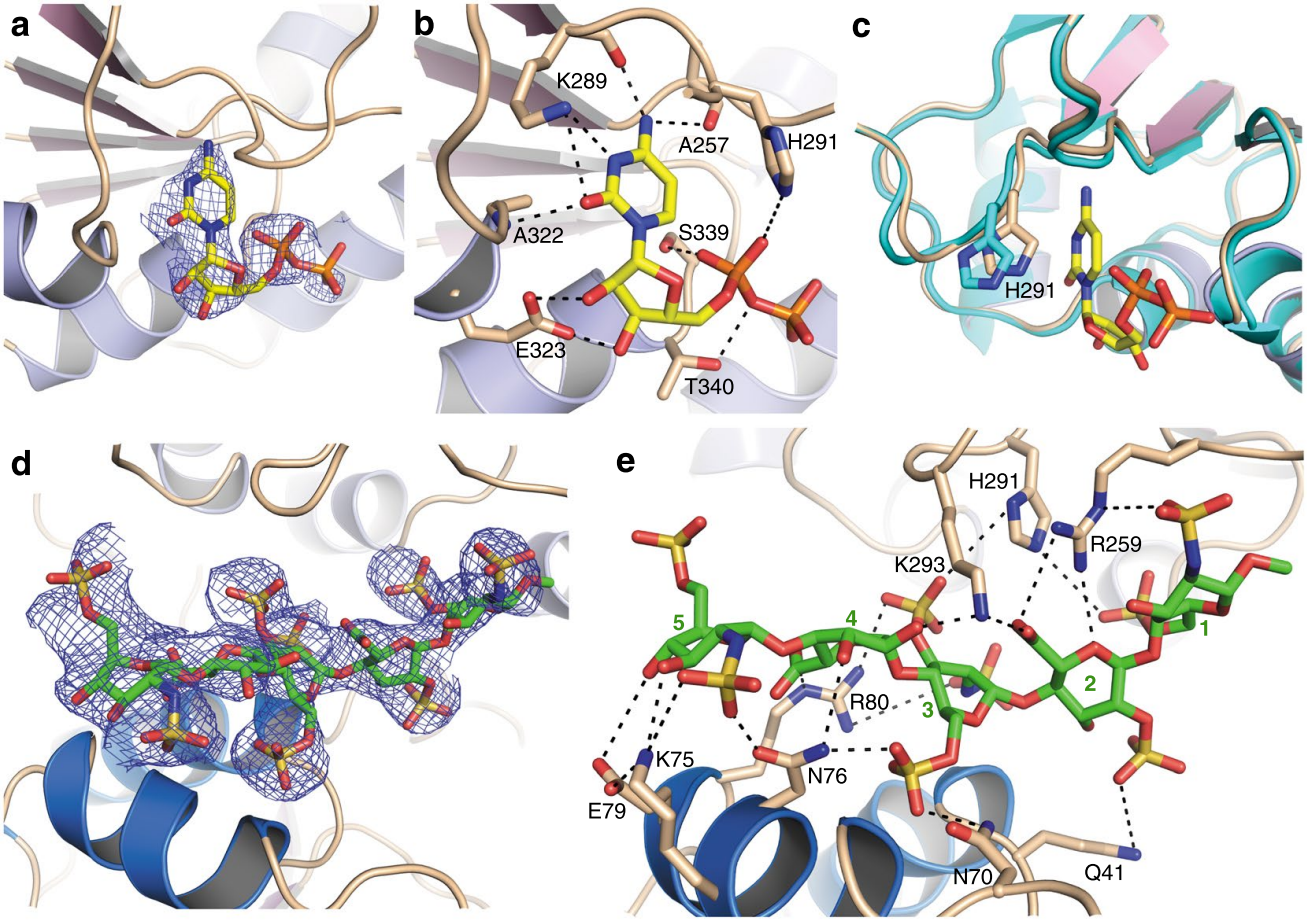
Structures of substrate-bound complexes of *Mh*PST. (**a**) Experimental Fo – Fc omit electron density map for CDP (yellow sticks) bound to *Mh*PST (ribbon diagram of monomer A) is shown as a blue mesh contoured at 3.0 σ. Structure factor calculation was performed prior to CDP docking. (**b**) Nucleotide donor binding site of *Mh*PST (monomer A) with residue side chains interacting with bound CDP (yellow sticks) shown in sticks and labelled. Potential hydrogen bonds are indicated as black dashes. (**c**) Superimposition of donor binding sites of the apo-structure (coloured in cyan) and of the CDP-bound structure (coloured in wheat, pink, and light blue). The shift of the side chain of H291 by 1.3 Å is shown. (**a**–**c**) The C-terminal domain is omitted for clarity. (**d**) Experimental Fo – Fc omit electron density map for fondaparinux (green sticks) bound to *Mh*PST (ribbon diagram of monomer A) is shown as a blue mesh contoured at 3.0 σ. Structure factor calculation was performed prior to ligand docking. (**e**) Acceptor substrate binding site of *Mh*PST (monomer A) with residue side chains interacting with bound fondaparinux (green sticks) shown in sticks and labelled. The five monosaccharide units of fondaparinux are labelled and potential hydrogen bonds are indicated as black dashes. (**a**,**d**) Electron density improved for all ligands upon refinement.

Attempts to obtain a structure with CMP-3FNeu5Ac, a non-hydrolyzable nucleotide activated sugar donor substrate derivative, were not successful. However, we were able to determine the structure of a binary complex with CDP at 3.0 Å resolution (Table 2). Clear electron density for the nucleotide diphosphate was observed, which allowed us to unambiguously model the CDP molecule in a cavity accessible from the cleft between the two Rossmann domains (Fig. 2a).

Although CDP binds at the interface of the two domains, it only makes extensive interactions with the C-terminal Rossmann domain (Supplementary Fig. S3), where the pyrimidine ring is inserted into a hydrophobic pocket formed by C256 and P292. The amine group (N4) of the cytosine base forms hydrogen bonds with the backbone carbonyl oxygen of K289 and A257, respectively. The side-chain amine of K289 also provides a hydrogen bond to the non-protonated N3 of the pyrimidine ring (Fig. 2b). This hydrogen-bonding network defines the specificity filter for CMP-activated sugar donor substrates, as none of these interactions would be possible for uracil or thymine bases. Furthermore, the binding pocket does not provide enough space to accommodate a purine base and does not facilitate any unspecific aromatic stacking interactions. However, the hydrogen bonding of the keto group (O2) of the cytosine base to the backbone amide of A322 and to the side chain amine of K289 could also occur with other nucleotides (Fig. 2b). All described interactions between *Mh*PST and cytosine highly resemble the situation observed for *Pasteurella multocida* mono-sialyltransferase PmST1 and related enzymes of the GT80 family^41–43^.

Binding of the ribose-phosphate moiety of CDP by *Mh*PST also exhibits a common interaction profile conserved amongst GT80 enzymes and lipooligosaccharide sialyltransferase from *N. meningitidis* representing the GT52 family (Fig. 2b)^41, 44^. In *Mh*PST, the O2′ and O3′ hydroxyl groups of the ribose are in close contact with the carboxyl group of E323 and form a strong bidentate hydrogen bond pair. The α-phosphate of CDP also makes extensive interactions with the C-terminal Rossmann domain. Phosphate oxygen O1 forms a hydrogen bond to the imidazole ring of H291, whereas O2 is hydrogen bonded by the hydroxyl group of S339. Additionally, the oxygen of the phosphate-phosphate bond connecting the α- and the β-phosphate forms a hydrogen bond to the hydroxyl group of T340. As the naturally occurring leaving group after the glycosyl transfer reaction is CMP and not CDP, T340 probably binds to the O3 oxygen of the α-phosphate in the native reaction. For the β-phosphate, the electron density is less well resolved (Fig. 2a) and no interactions with the protein are observed. This is also reflected by its altered conformation in the two *Mh*PST monomers of the asymmetric unit. While the two CMP moieties of CDP superimpose very well (r.m.s.d. = 0.29 Å), the β-phosphate in monomer B is flipped by 140° as compared to the orientation in monomer A (which is presented in Fig. 2a–c) and points towards H291.

All residues forming specific side-chain interactions with CDP are conserved among bacterial PSTs (Supplementary Fig. S4), suggesting that the donor nucleotide-binding site exhibits identical features in other GT38 enzymes. H291 is not only invariant in bacterial PSTs, but is part of the HP-motif generally conserved in sialyltransferases, as well as in β-Kdo transferases^24, 33, 45^. Structure-function studies on PmST1 proposed that H311 (H291 in *Mh*PST) is involved in stabilizing the negatively charged CMP leaving group^46^. Indeed, mutation H291A in *Mh*PST resulted in a seven-fold increased K_m_ for the CMP-Neu5Ac donor substrate and a 38-fold reduced k_cat_ (280-fold reduced catalytic efficiency, k_cat_/K_m_), suggesting a significant role in catalysis (Table 1). As expected, the K_m_ for the acceptor substrate Sia_2_Lac is only marginally affected by the H291A mutation (Table 3).

To our surprise, CDP binding did not result in major conformational changes in the *Mh*PST structure (r.m.s.d. = 0.41 Å over 2802 atoms), and the most significant difference is the movement of the H291 imidazole side chain towards the bound CDP ligand by 1.2 Å (distance between the Nε^2^ atoms of H291 in the two different conformations; Fig. 2b). This is in strong contrast to other GT-B enzymes, where nucleotide binding usually causes large domain movements in creation of the donor sugar-binding site. In PmST1 for example, CMP binding results in a 23° rotation of the N-terminal Rossmann domain towards the C-terminal domain, thereby closing the catalytic cleft (Supplementary Fig. S6a)^41^. However, in a recent study on β-Kdo transferase of GT-family 99 that is related to sialyltransferases, CMP binding also did not induce any substantial domain movements^24^, suggesting that nucleotide binding might not always be sufficient to trigger these rearrangements.

### Complex structure with fondaparinux reveals acceptor-binding site

As discussed above, our attempts to crystallize *Mh*PST in the presence of the di-sialylated acceptor substrate Sia_2_LacNAc6S did not resolve the ligand in the crystal structure. However, we could obtain a complex with fondaparinux, a synthetic polyanionic (heparin) pentasaccharide clinically used as an anticoagulant (Table 2, Supplementary Fig. S5b). Clear electron density was observed only in monomer A, which allowed us to unambiguously model the ligand into electron density lining the deep catalytic cleft between the two Rossmann domains (Fig. 2d). Based on the repeating polyanionic functional groups, we propose fondaparinux maps on to *Mh*PST in a complementary electropositive path similar to that required of the native polySia substrate. We note, however, that fondaparinux carries an additional five anionic functional groups compared to a polySia pentamer and would also be expected to span a comparatively shorter distance than a corresponding nine-carbon sugar sialic acid pentamer would do (Supplementary Fig. S5).

**Figure 3:**
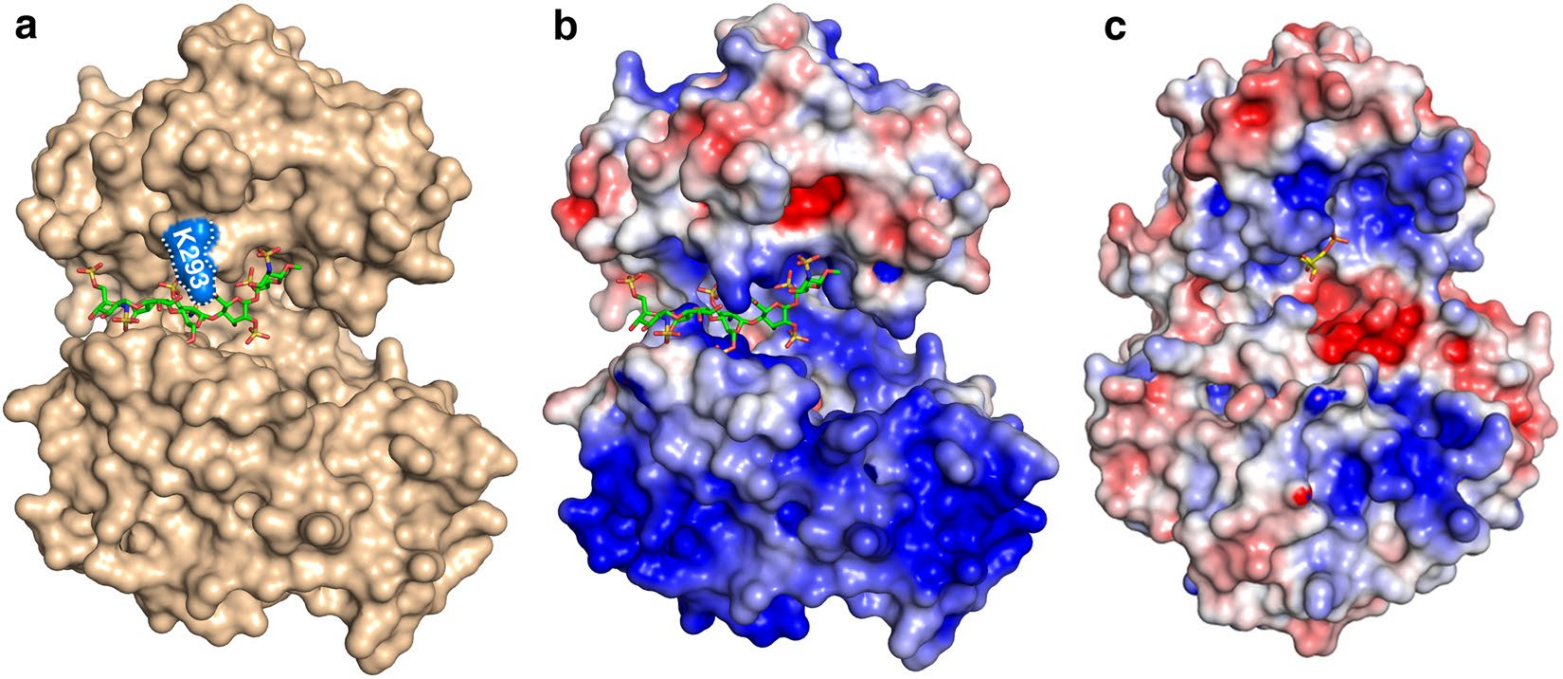
The *Mh*PST acceptor substrate binding groove at the interface of the N-terminal and the C-terminal domain. (**a**) Surface representation of *Mh*PST (monomer A in wheat) with bound acceptor ligand analogue fondaparinux shown in green sticks. The surface of residue K293 is coloured in blue and is labelled. (**b**) Electrostatic surface potential of fondaparinux bound *Mh*PST structure. (**c**) Electrostatic surface potential of CMP bound structure of mono-sialyltransferase PmST1 (PDB: 3s44). (**b**,**c**) Surface potentials were calculated with the Adaptive Poisson Boltzmann Server^69, 70^ with a PARSE force field, with linear interpolation of colours at intermediate potentials (blue, 4 kT/e; red, −4 kT/e; white, 0 kT/e; probe radius, 1.4 Å).

Fondaparinux is bound to *Mh*PST along an electropositive groove (Fig. 3b) by a series of interactions, whereby both carboxyl groups and seven out of the eight sulfate groups are at least partly liganded. In contrast to CDP binding which only involves the C-terminal domain, both Rossmann domains contribute to the fondaparinux binding site (Fig. 2e). Notably, the 6′ carboxyl group of the second saccharide (IdoA2S) is saturated by interactions with the guanidinium group of R259 and the terminal amine of K293. R259 further forms a salt bridge to the 2′ sulfoamino group of the reducing end saccharide (GlcNS6S-OMe). The amine group of K75 on the other side binds to the 2′ sulfoamino group of the non-reducing end saccharide (GlcNS6S), whereas the side-chain of R80 interacts with the 6′ carboxyl group of the fourth saccharide (GlcA), as well as with the 2′ sulfoamino group and the 3′ sulfo group of the third sugar (GlcNS3,6 S) (Fig. 2e). Residues K75, R259, and K293 are strictly conserved in bacterial PST enzymes (Supplementary Fig. S4), suggesting that they also play an important role in binding the natural polySia acceptor substrate.

A surface representation of *Mh*PST further illustrates the central role of K293 in acceptor substrate binding. Its side chain reaches deeply into the catalytic cleft, thereby pinning the bound fondaparinux against the N-terminal domain and locking it in the resulting cavity (Fig. 3a). K293 contacts the pentasaccharide between the second and the third sugar residue, which might explain the preference for tri-sialylated over di-sialylated acceptor substrates (Table 3)^34^. These observations were corroborated by mutagenesis studies, where mutant K293A had a drastically reduced catalytic efficiency, while the K_m_ value for the CMP-Neu5Ac donor substrate was not negatively affected (Tables 1, 3).

Superimposition of the ligand-free and the fondaparinux-bound structures showed that fondaparinux binding does not cause a movement between the N- and the C-terminal domains (r.m.s.d. = 0.66 Å over 2889 atoms). The observed small conformational rearrangements (<2.0 Å) are primarily located in the catalytic cleft and are required to accommodate the fondaparinux ligand.

### Catalytic mechanism of bacterial PST

**Figure 4:**
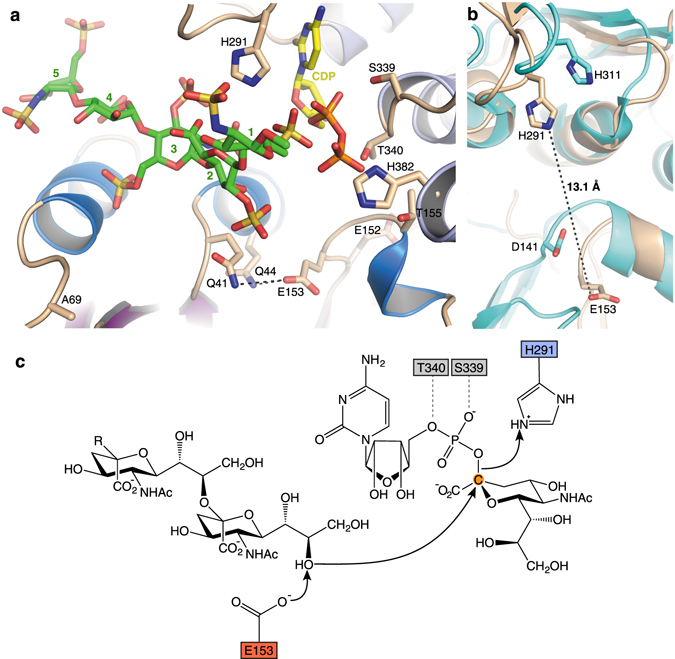
*Mh*PST active site and identification of the catalytic base. (**a**) Modelled ternary complex by superimposition of the CDP-bound structure and the fondaparinux-bound structure. *Mh*PST (monomer A) is shown as a ribbon diagram with the same color-coding as in Fig. 1c, CDP is shown as yellow sticks, fondaparinux is shown as green sticks and the five monosaccharide units are indicated. Residues with a supposed role in catalysis are shown in sticks and are labelled. Potential hydrogen bonds between E153 and Q41 and Q44, respectively are indicated as black dashes. The location of the mutation K69A is indicated. (**b**) Superimposition of active site of *Mh*PST (wheat ribbon diagram) and mono-sialyltransferase PmST1 in open conformation (PDB: 3s44, cyan ribbon diagram). The catalytic acid and base are shown in sticks and are labelled for both enzymes, and the distance between E153 and H291 is indicated for *Mh*PST. (**c**) S_N_2-like reaction mechanism of *Mh*PST. The catalytic base E153 (red box) abstracts a proton from the C8′ hydroxyl group of the sialic acid acceptor concerted with the nucleophilic attack on the anomeric C2′ carbon (orange circle) of the CMP sialic acid donor substrate, thereby generating an α-2,8 glycosidic linkage. The resulting negatively charged CMP leaving group is stabilized by H291 (blue box) assisted by S339 and T340 (grey boxes). R = α-2,8-linked oligosialyl.

To generate a modelled composite of a ternary complex for mechanistic analysis, we superimposed the CDP-bound and the fondaparinux-bound structures (Fig. 4a). We propose that the resulting complex resembles a pseudo product complex, in which the reducing end sugar of fondaparinux is in proximity to the α-phosphate of the CDP. We note, however, that the native polySia acceptor binds in the opposite direction with the non-reducing end sugar oriented close to the sugar nucleotide donor (Supplementary Fig. S5). The functional analysis of residue R259, which interacts with the 2′ sulfoamino group of the reducing end saccharide of fondaparinux, supports this hypothesis. Mutant R259A showed a 3-fold increased K_m_ for the CMP-Neu5Ac donor, while the K_m_ for the Sia_2_Lac acceptor was not affected (Tables 1, 3). Therefore, R259 could, under natural reaction conditions, potentially interact with the sialic acid moiety of the CMP-Neu5Ac donor, which gets transferred to the non-reducing end of the growing polySia chain.

Sialic acid transfer occurs with inversion of configuration (from the β-linked CMP-Neu5Ac donor to the α-2,8-linked polySia), and PST has been proposed to follow a S_N_2-like direct displacement mechanism^45^. While H291 could act as a catalytic acid to stabilize the nucleotide phosphate-leaving group, the catalytic base remained unknown. The analysis of the *Mh*PST ternary complex structure did not reveal any residue in proximity to the active site that could serve this purpose. We therefore performed a comparison of our *Mh*PST structure with structures of the PmST1 enzyme, where the catalytic base had been identified^46^. As described before, PmST1 undergoes large domain movements upon nucleotide donor substrate binding resulting in a closure of the catalytic site (Supplementary Fig. S6a). As a consequence, the distance between the catalytic base (D141) and residue H311 changes from 14.0 Å in the open conformation to 8.4 Å in the closed state (Supplementary Fig. S6b). Structural alignments between *Mh*PST and PmST1 not only indicated that our *Mh*PST structures resemble an open conformation, but also identified residue E153 as a potential catalytic base. E153 is located in an analogous loop to D141 in the PmST1 structure and is 13.1 Å away from residue H291 (Fig. 4b). Furthermore, E153 is part of a previously described D/E-D/E-G motif and is conserved in bacterial PSTs (Supplementary Fig. S4)^33, 45^. Kinetic analyses of *Mh*PST mutants confirmed that E153 is indeed the catalytic base with an activity too low to assign a K_m_ value to it. The neighbouring mutant E152A was a 300-fold worse catalyst, suggesting that this adjacent residue plays an important role in stabilizing the catalytic loop between strand β5 and helix α5a (Tables 1,3, Fig. 4a). We therefore propose a reaction mechanism for *Mh*PST, in which the carboxyl group of E153 abstracts a proton from the C8′ hydroxyl group of the non-reducing end sialic acid of the acceptor substrate (Fig. 4c) in concert with attack on the anomeric C2′ carbon of the CMP-Neu5Ac donor substrate, forming an α-2,8 glycosidic linkage between the two sialic acid residues. H291 acts as a catalytic acid to stabilize the negative charge at the terminal phosphate of the CMP leaving group. Residues S339 and T340 might further assist in the coordination of the phosphate. Residues Q41 and Q44 which are in proximity to E153 might play a role in activating the catalytic base as reflected by the reduced catalytic performance of mutants Q41A and Q44A (Tables 1, 3, Fig. 4a,c).

## Discussion

The structural characterization of *Mh*PST in the presence of the donor substrate analogue CDP and the acceptor substrate mimetic fondaparinux revealed the molecular concepts of substrate binding and catalysis. Residues involved in these interactions are generally conserved among PSTs from different species, despite an overall sequence identity between 28% and 31% (Supplementary Fig. S4) making *Mh*PST a highly valuable model to study the catalytic mechanism of bacterial PSTs. Notably, substrate binding did not cause any significant rotation of the N- and C-terminal Rossmann domains that is frequently observed in other GT-B enzymes, and which leads to a closure of the catalytic cleft. A structural comparison with the mono-sialyltransferase PmST1 revealed that *Mh*PST adopts an open conformation (Fig. 4b). We suggest this may be a reflection of the lack of complete donor substrate CMP-Neu5Ac bound (see below), or the need to accommodate the larger, polymeric PST acceptor substrate. Even though binding of the substrate mimetic fondaparinux did not provoke domain closure in *Mh*PST (perhaps because it is too short or because the precise alignment of sugar units is incompatible with triggering closure), a rotation of the N-terminal domain appears to be necessary to move the proposed catalytic base E153 up to the active site and to position its carboxyl group appropriately to activate the C8′ hydroxyl group of the sialic acid acceptor for nucleophilic attack (Fig. 3c). Therefore, we postulate the existence of *Mh*PST in a closed state during the catalytic cycle. What could be the trigger for such a conformational change? In the case of the mono-sialyltransferase PmST1, CMP binding induced an N-terminal domain movement, in which S143 located in helix α5a (and in proximity to the catalytic base D141) moves 5.2 Å towards the C-terminal domain to form a hydrogen bonding network with Y388 and the terminal phosphate of CMP. Furthermore, the side chain of Y388 is flipped by 180° and the corresponding helix α12b is shifted by 5.5 Å upon CMP binding^41^. *Mh*PST contains the conserved residues T155 and H382 at corresponding positions that could emulate the function of S143 and Y388 in PmST1, respectively (Fig. 4a, Supplementary Fig. S4). As CDP binding did not facilitate these interactions in *Mh*PST, it is important to note that bound CMP in the complex structure of PmST1 resulted from hydrolysis of the complete donor substrate CMP-Neu5Ac^41^, and it cannot be excluded that the domain shift occurred before donor hydrolysis. Therefore, binding of the complete sugar donor substrate (or its non-hydrolysable derivative CMP-3FNeu5Ac) might be required to induce the domain shift.

The open conformation of *Mh*PST exhibits a deep cleft between the two Rossmann domains spanning across the entire front of the enzyme. This electropositive groove (~35 Å in diameter) is much more pronounced than in other glycosyltransferases bearing a GT-B fold (Fig. 3b,c) and is concordant with accommodating a polymeric and polyanionic sialic acid acceptor substrate. Interestingly, saturation transfer difference NMR spectroscopy studies on PST from *N. meningitidis* serotype B (*NmB*PST) postulated the existence of an extended acceptor-binding site that can accommodate at least six sia residues^47^. Binding of the acceptor mimetic fondaparinux to *Mh*PST illustrates that the ligand is well aligned between the two domains already in the observed open conformation (Fig. 3a). The formation of the postulated closed enzyme state during catalysis would cause an even more snug fit of the acceptor substrate in the catalytic cleft. These acceptor-binding properties may suggest a processive mechanism of polymerization, in which the growing polySia chain is retained at the active site for addition of multiple sia monomers before product release. However, several *in vitro* studies using purified PST enzyme proposed a distributive mechanism, where polySia is released from the enzyme after each transfer reaction^33, 38^. *In vitro* studies on PST are generally performed on soluble enzyme variants and utilize soluble synthetic acceptor substrates resulting in a reduced local concentration of acceptor substrate, because PST as well as the lipid-linked polySia acceptor are naturally anchored in the inner membrane (Fig. 1a). Therefore, it is not surprising to observe a discrepancy between polySia polymer length *in vivo* and *in vitro* ^26, 48, 49^. A recent study on the polySia product profile of *NmB*PST proposed that chain elongation *in vitro* occurs in an abortive processive manner with frequent dissociation of the enzyme-acceptor complex^50^. Increasing acceptor length resulted in increased enzyme affinity suggesting a continuous binding site able to interact with a 20-mer polySia acceptor. Intriguingly, the authors identified residue K69 as a molecular switch controlling the mechanism of chain elongation and polySia size distribution. Mutations K69Q and K69D changed the chain elongation to a distributive mechanism, yielding reduced product dispersity even for short oligoSia acceptors and a direct interaction of residue 69 with the substrate was proposed^50^. K69 is also conserved in *Mh*PST (Supplementary Fig. S4), but in order to obtain well-diffracting crystals, it was mutated to alanine. The distance between the methyl group of A69 and the second or third fondaparinux saccharide (IdoA2S or D-GlcNS3,6 S) is more than 10 Å suggesting that even a lysine residue at position 69 would require a domain closure to directly interact with the acceptor substrate (Fig. 4a). However, we cannot exclude that the presence of K69 in *Mh*PST would result in increased acceptor binding. Additional sites of mutation in *NmB*PST that were found to influence polySia size distribution are not conserved among bacterial PSTs. Even though the effect of these other mutations seems to be specific for *NmB*PST, the surface potential of *Mh*PST shows two highly electropositive areas at the front of the N-terminal Rossmann domain, which could provide additional interaction surfaces for an extended polySia chain (Fig. 3b). For comparison, the surface of the mono-sialyltransferase PmST1 is lacking a pronounced acceptor-binding groove and mainly shows positive values for the donor-binding site, concordantly with the preference for short uncharged acceptor substrates (Fig. 3c). Therefore, the two positively charged patches on the surface of *Mh*PST could represent an extension of the acceptor-binding groove providing a large interaction interface with low site-specific binding but high avidity for the growing polySia chain. Such a model for acceptor binding would allow substrate translocation from one site to the next and would be in excellent agreement with the higher affinity for long acceptor oligomers observed for *NmB*PST^50^.

Strikingly, an analogous mechanism for substrate interaction has been brought forward for mammalian PSTs. The structure of the human ST8SiaIII enzyme also exhibits an extensive positively charged surface groove able to accommodate extended polySia acceptor substrates^35^. Apart from this conceptual similarity in polySia binding, mammalian and bacterial PSTs share no common features. The two enzymes exhibit completely different folds and none of the conserved motifs defining the active site of mammalian PSTs and of other eukaryotic mono-sialyltransferases of the GT29 family are found in bacterial *Mh*PST belonging to the GT38 family^35, 51–53^. Instead, the molecular principles of substrate binding and catalysis of bacterial PSTs resemble enzymes of CAZy families GT52 and GT80^33, 44, 46^. Therefore, polySia biosynthesis is a prototype of convergent evolution where bacterial and mammalian enzymes follow different molecular routes to synthesize the identical α-2,8-linked polySia homopolymer. Since polySia biosynthesis is an essential virulence factor for the corresponding pathogens, bacterial PST might be an interesting target for the development of novel antibiotics. Due to the lack of structural similarity between the bacterial and mammalian PSTs shown for the first time here, our insights into *Mh*PST provide encouragement regarding the ability to create bacterial PST-specific therapeutics.

The biosynthesis of polySia has also great potential for various medical applications, and both bacterial and mammalian PSTs represent potential candidates to produce polySia and polysialylated bioconjugates. The broad application of mammalian PSTs is currently limited by their high acceptor protein specificity^2, 11, 54^, whereas bacterial PSTs exhibit a more relaxed substrate specificity^26^. Different bacterial enzymes including *Mh*PST have been successfully used to polysialylate a primed version of fetuin as well as different cell surface proteins including NCAM, the most prominent acceptor protein for mammalian PSTs^34, 55^. Furthermore, an elegant two-step enzymatic polysialylation strategy was applied to site-specifically modify alpha-1-antitrypsin resulting in improved pharmacokinetic properties^56^. These data illustrate the tremendous value of using bacterial PST enzymes for different therapeutic applications. Our structural characterization of the *Mh*PST enzyme might therefore contribute to the development of specific and tailored polySia-conjugates.

## Methods

### Cloning and expression of Δ20*Mh*PST

The polysialyltransferase gene from *M. haemolytica A2* was cloned as a Δ20 N-terminal truncation into the pCW expression vector as previously described^34^. To obtain well-ordered crystals, two surface entropy reduction mutations (K68A, K69A) were introduced. This double mutant was referred to as wild-type enzyme and all further mutations in the active site that were used for kinetic studies were based on it. All mutations were introduced by site directed mutagenesis using the Quick Change method.

Δ20*Mh*PST constructs were transformed into *E. coli* AD202 cells and a single clone was used to inoculate a preculture in LB media supplemented with 100 µg/mL ampicillin. The main culture of 2xYT media supplemented with 100 µg/mL ampicillin was inoculated to an OD600 of 0.05 and cells were grown at 37 °C and 200 rpm until an OD600 of 0.4 to 0.6 was reached. The culture was shifted to 20 °C and expression was induced by addition of 0.5 mM IPTG at an OD600 of 0.8. After incubation for 16 h at 20 °C and 200 rpm, cells were harvested by centrifugation and cell pellets were stored at −80 °C until use.

### Purification and crystallization of Δ20*Mh*PST

5 g of frozen cells were resuspended in 25 mL of buffer A consisting of 50 mM HEPES, pH 7.4; 150 mM NaCl; 5 mM β-mercaptoethanol and Complete Mini protease inhibitor cocktail (Roche). Cells were lysed by French Press (2 passes at 1,500 psi) and cell debris were removed by centrifugation at 48,000 × g for 30 min. The supernatant was passed through a filter with 0.45 μm pore size, before the sample was loaded onto a 5 mL Heparin HP column (GE Healthcare) equilibrated with buffer A. The column was washed with 5 column volumes of buffer A, followed by a second wash with 5 column volumes of 15% buffer B consisting of 50 mM HEPES, pH 7.4; 1.5 M NaCl and 5 mM β-mercaptoethanol. The protein was eluted in a linear gradient of 0–100% buffer B over 6 column volumes and 1 mL elution fractions were collected. Fractions containing *Mh*PST were pooled and the buffer was immediately exchanged to buffer C consisting of 50 mM HEPES, pH 7.2 and 100 mM NaCl using a HiPrep desalting column (GE Healthcare). Protein purity was evaluated by SDS-PAGE, and the protein concentration was determined by absorption at 280 nm using an extinction coefficient of 37250 M^−1^cm^−1^. Protein monodispersity was analysed by analytical size exclusion chromatography using a Superdex 200 column (GE Healthcare, eluent buffer C). All steps were performed at 4 °C.

For crystallization, the protein was concentrated to 4–5 mg/mL using an Amicon centricon with a molecular weight cut-off of 30 kDa. Initial Δ20*Mh*PST crystals were observed by vapour diffusion in sitting drops under conditions containing PEG 3350. Crystallization conditions were optimized to 17–24% PEG3350 (v/v); 140–250 mM Mg_2_SO_4_ and 100 mM MES, pH 7.2 in a 1:1 drop ratio using the microbatch method. Crystals appeared after 1–2 h at 23 °C and grew to final size within 2 days. Δ20*Mh*PST was co-crystallized with 5 mM CDP donor analogue, or 2 mM Sia_2_LacNAc6S acceptor analogue in the same conditions. Apo crystals were soaked with 2 mM fondaparinux in 200 mM MgSO_4_; 100 mM MES, pH7.2 and 20% PEG3350 (v/v) for 2 h by transferring crystals to the soaking solution.

### Data collection, phasing and refinement

Δ20*Mh*PST crystals were cryoprotected in 200 mM MgSO_4_; 100 mM MES, pH7.2; 20% PEG3350 (v/v) and 30% glycerol (v/v) and flash frozen in liquid nitrogen. For phasing, 500 mM sodium bromide was added to the cryoprotectant. X-ray diffraction data were collected at both the Advanced Light Source (beamline 5.0.2) and the Canadian Light Source (CMCF beamlines 08ID-1 and 08B1–1). Data were integrated with XDS^57^ and scaled and merged with Aimless^58^. Phases for the bromide derivative crystals were solved by SAD using autoSHARP^59^ and further density modification was carried out using PHENIX^60^. The initial model was further built manually in Coot^61^ and refined with REFMAC^62, 63^ and PHENIX^60^. Co-crystal structures of Δ20*Mh*PST in complex with Sia_2_LacNAc6S, CDP, or fondaparinux were solved by molecular replacement with the apo structure of Δ20*Mh*PST using PHASER^64^. Processing and refinement statistics are summarized in Table 2. (The structures of Δ20*Mh*PST apo, Δ20*Mh*PST + Sia_2_LacNAc6S and Δ20*Mh*PST + fondaparinux contain R132 as Ramachandran outlier.) The topology diagram was created with PDBsum^65^ and all structure images were created with PyMOL^66^.

### *In vitro* polysialylation activity assay

The polysialylation activity of purified *Mh*PST batches was tested in an *in vitro* reaction containing 0.5 mM BODIPY-diSiaLac acceptor; 10 mM CMP-Neu5Ac donor; 50 mM HEPES, pH 7.4; 10 mM MgCl_2_ and 0.2 mg/mL enzyme in a total volume of 10 μl. The reaction was incubated at 37 °C for 16 h, before 1 μl of the reaction was applied to a silica gel 60 TLC plate and the sample was separated with a developing phase containing ethylacetate:methanol:H_2_O:acetic acid in a ratio of 4:2:1:0.1. TLC plates were illuminated under UV light to visualize acceptor substrate conversion.

### Chemo-enzymatic synthesis of disialyllactose (Sia_2_Lac) and trisialyllactose (Sia_3_Lac)

Both oligosaccharides (Sia_2_Lac/Sia_3_Lac) were chemo-enzymatically prepared using bifunctional Cst-II (from *Campylobacter jejuni*) as previously described^67^. In brief, 118 mg of lactose (Sigma Aldrich) and 210 mg of CMP-Neu5Ac (Roche) were dissolved in 9 mL of 100 mM Tris, pH 7.9 containing 20 mM MgCl_2_ at room temperature. The reaction was initiated by addition of 220 μL of Cst-II (stock: 12.5 mg/mL) and 10 µL alkaline phosphatase (Sigma Aldrich) in order to degrade liberated CMP. The reaction was incubated at 25 °C and another 210 mg of CMP-Neu5Ac were added after 2 h and 18 h, respectively. The pH was carefully monitored and adjusted with NaOH as needed. Reaction progress was monitored by TLC (EtOH:n-BuOH:pyridine:H_2_O:AcOH = 100:10:10:30:3) and the reaction was stopped with 3 mL ice-cold EtOH, centrifuged (10 min at 4,000 × g) and the supernatant was applied to an Amicon centricon with a molecular weight cut-off of 3 kDa to remove remaining protein. Sia_2_Lac and Sia_3_Lac were group separated using P-2 size exclusion chromatography (BioRad, eluent: 20% EtOH) and EtOH was removed *in vacuo*. After MacroQ anion exchange chromatography (GE Healthcare, flow rate: 2 mL/min, gradient: 0% to 100% of 0.4 M ammonium formate in 80 min), Sia_2_Lac and Sia_3_Lac fractions were pooled separately, the volume was reduced and both products were ran again on a P-2 column to exchange the buffer to 20% EtOH. Only highly pure fractions were pooled, lyophilized and products were confirmed by ESI mass spectrometry. Final yields were determined with 23% Sia_2_Lac and 28% Sia_3_Lac.

### Kinetic studies

Kinetic parameters of *Mh*PST mutants were determined with a coupled enzyme assay as described previously^68^. Briefly, assays were carried out in 96-well plates (half-area wells, Corning) with varying substrate concentrations of Sia_2_Lac/Sia_3_Lac (with constant concentration of CMP-Neu5Ac), or CMP-Neu5Ac (with constant concentration of colominic acid as acceptor). Each well contained 100 μL 50 mM HEPES, pH 7.0; 50 mM KCl; 20 mM MgCl_2_; 1 mg/mL BSA; 2 mM ATP; 2 mM phosphoenolpyruvate; 1 mM NADH; 16 U/mL pyruvate kinase; 29 U/mL lactate dehydrogenase; 0.07 U/mL nucleoside monophosphate kinase (Roche) and appropriate concentration of enzyme mutants. Prior to the addition of *Mh*PST, the assay mixture was incubated at 37 °C until a stable baseline in absorbance at 340 nm (NADH) was achieved. Once *Mh*PST enzyme was added, the rate of NADH consumption was determined by measuring the continuous decrease in absorbance at 340 nm. Rates were converted by using a NADH standard curve in order to calculate k_cat_ (s^−1^). An extinction coefficient for NADH of 6,300 M^−1^cm^−1^ was used for calculations. Since two equivalents of NADH were released per equivalent of CMP-Neu5Ac consumed, the rate of transfer was determined as half the rate of NADH consumption.

### Accession codes

Atomic coordinates and structure factors for Δ20MhPST apo, Δ20MhPST + CDP, Δ20MhPST + Sia_2_LacNAc6S and Δ20MhPST + fondaparinux have been deposited in the Protein Data Bank under PDB codes 5WC﻿8, 5WCN, 5WC6 and 5WD7, respectively.

## Electronic supplementary material

Supplementary Information
