# Supplementary material for: X-ray crystallographic structure of a bacterial polysialyltransferase provides insight into the biosynthesis of capsular polysialic acid
Source: Sci Rep. 2017 Jul 19;7:5842. doi: 10.1038/s41598-017-05627-z (PMC5517516; doi:10.1038/s41598-017-05627-z)
Supplement: Supplementary file 1 — Supplementary Information [file 41598_2017_5627_MOESM1_ESM.pdf]

SUPPLEMENTARY INFORMATION

X-ray crystallographic structure of a bacterial polysialyltransferase provides insight into the biosynthesis of capsular polysialic acid

Christian Lizak[1,2,6], Liam J. Worrall[1,2], Lars Baumann[3], Moritz Pfleiderer[1,2], Gesa Volkers[1,2], Tianjun Sun[1,2], Lyann Sim[5], Warren Wakarchuk[5], Stephen G. Withers[3,4], Natalie C.J. Strynadka[1,2 *]

[1]Department of Biochemistry and Molecular Biology, University of British Columbia, Vancouver, BC V6T 1Z3, Canada.

[2]Centre for Blood Research, University of British Columbia, 2350 Health Sciences Mall, Vancouver, BC, V6T 1Z3, Canada

[3]Department of Chemistry, University of British Columbia, Vancouver BC V6T 1Z1, Canada

[4]Department of Biochemistry and Molecular Biology, Centre for High-Throughput Biology, University of British Columbia, Vancouver BC V6T 1Z4, Canada

[5]Department of Chemistry and Biology, Ryerson University, Toronto ON M5B 2K3, Canada

[6]Current address: LimmaTech Biologics AG, Grabenstrasse 3, 8952 Schlieren, Switzerland

*Corresponding author E-mail: ncjs@mail.ubc.ca

Supplementary results

Supplementary Figure S1

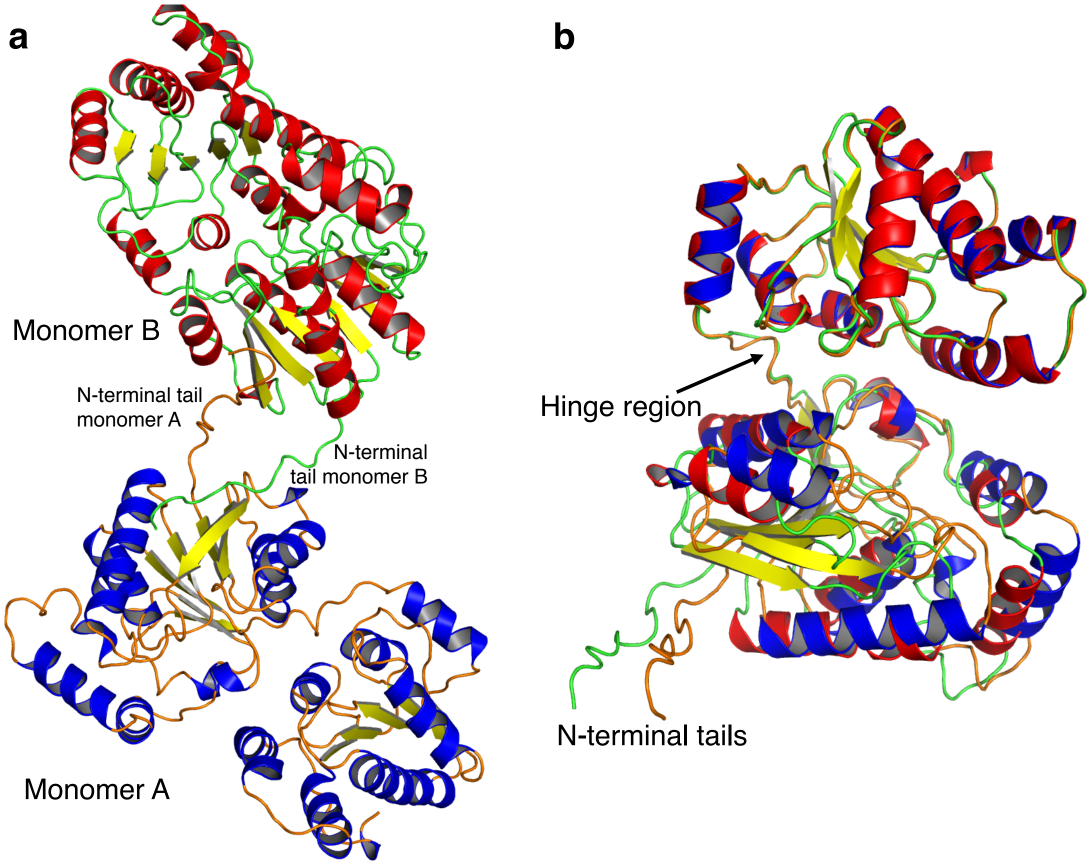

Supplementary Figure S1 | Ribbon drawing of Δ20*Mh*PST. (a) Two *Mh*PST monomers are present in the asymmetric unit. In monomer A, the backbone is coloured in orange, helices are in blue, and β-sheets are in yellow. In monomer B, the backbone is coloured in green, helices are in red, and β-sheets are in yellow. The N-terminal tails are indicated. (b) Superimposition of the two *Mh*PST monomers based on their C-terminal domain. The colour coding is the same as in (a) and the hinge region between the N-terminal and the C-terminal Rossmann fold is indicated.

Supplementary Figure S2

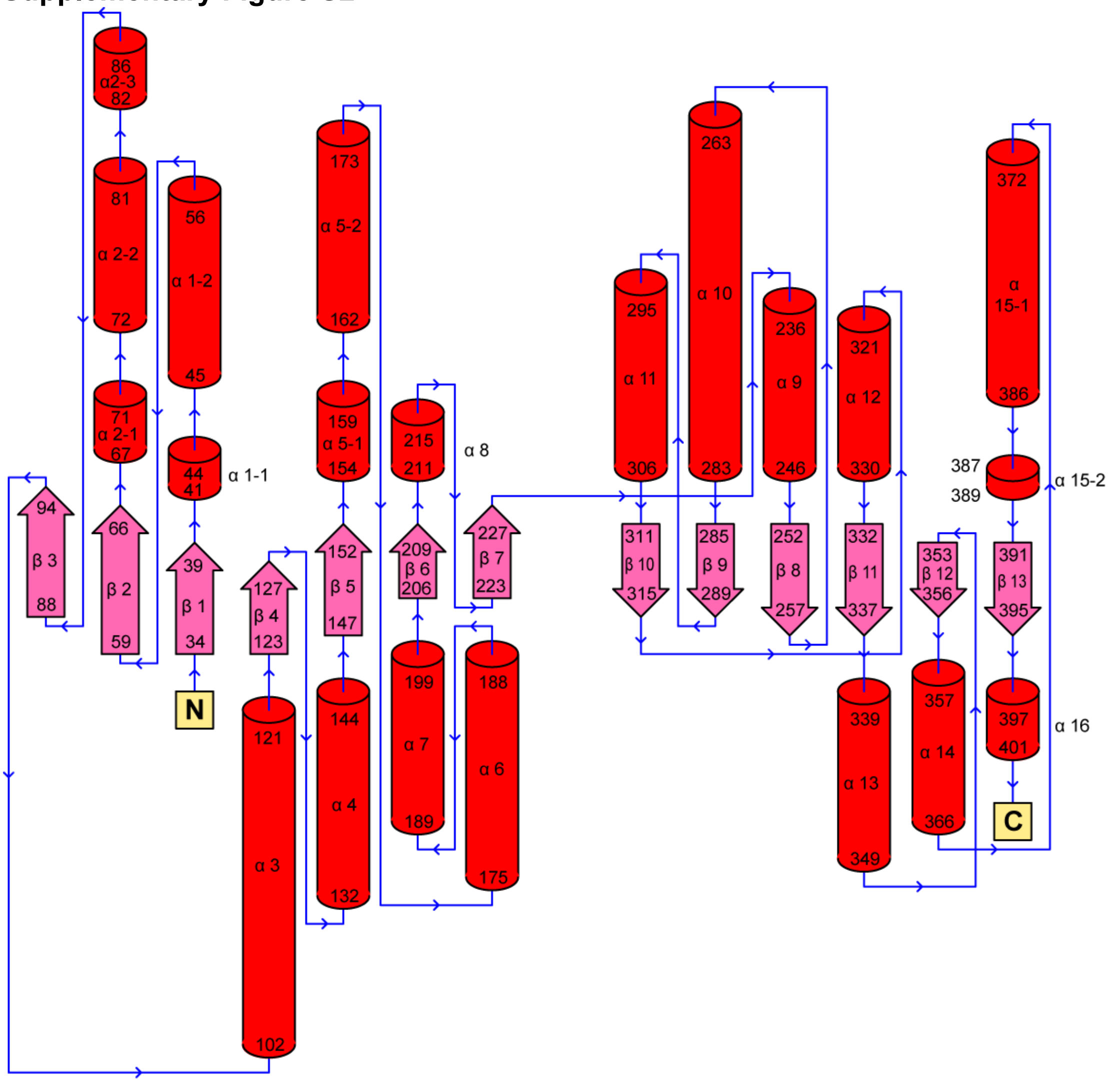

Supplementary Figure S2 | Topology diagram of Δ20*Mh*PST. α-Helices are shown as cylinders, β-sheets are shown as arrows and the numbers for individual secondary structure elements are indicated. The diagram was created with PDBsum (https://www.ebi.ac.uk/pdbsum/).

Supplementary Figure S3

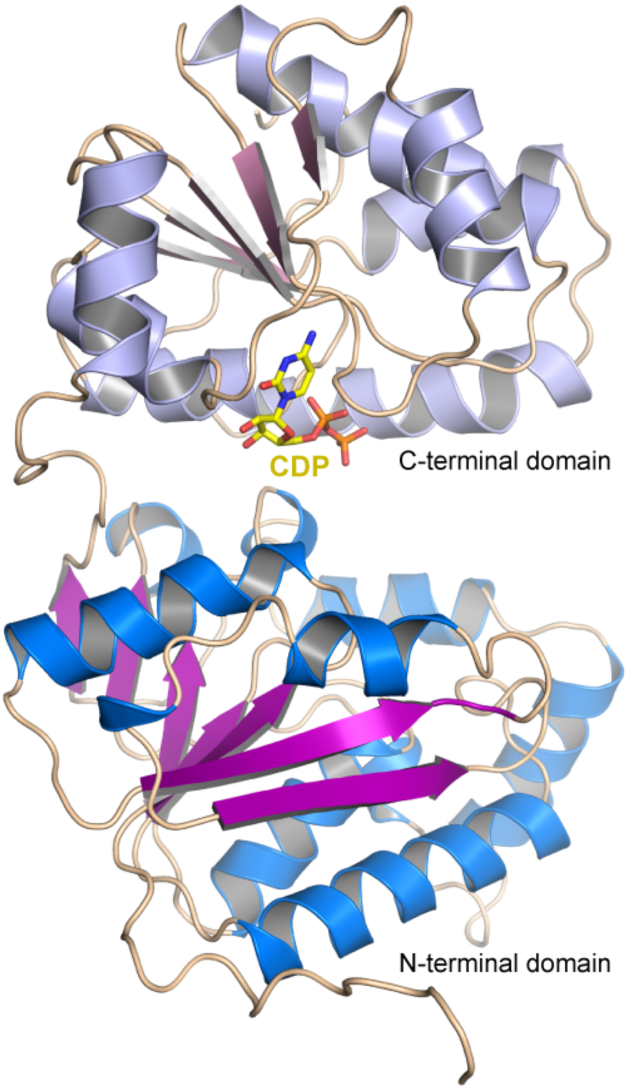

Supplementary Figure S3 | Ribbon diagram of the CDP-bound Δ20*Mh*PST structure. In the N-terminal Rossmann domain, helices are coloured in marine and β-sheets are coloured in purple, whereas in the C-terminal Rossmann domain, helices are coloured in light blue and β-sheets are coloured in pink. Bound CDP is shown as sticks and is labeled.

Supplementary Figure S4

```
 M. haemolytica A2  M-----IKTIKKLLVSPIEFFQDSWFLKFH------LAEDYRKTTNLFFI
 E. coli_K1         MIFDASLKKLRKLFVNPIGFFRDSWFFNSKNKAEELLSPLKIKSKNIFII
 N. meningitidis B  M-----LKKIKKALFQPKKFFQDSMWLTTS------PFYLTPPRNNLFVI
                    *     :*.::* :..*  **:** ::.             .*:*.*

                     Q41 Q44                     69KN70  K75  E79
 M. haemolytica A2  SQMGQLEQYQGLIEKLKLKNNVLIVLYTKKNQLMPKNIAERCNKELFNSI
 E. coli K1         SNLGQLKKAESFVQKFSKRSNYLIVLATEKNTEMPKIIVEQINNKLFSSY
 N. meningitidis B  SNLGQLNQVQSLIKIQKLTNNLLVILYTSKNLKMPKLVHQSANKNLFESI
                    *::***:: :.:::  .  .* *::* *.**  *** : :  *::**.*

 M. haemolytica A2  RFLCLPKSPMRLNIKNYIMMLNSYKLLLKRIKPKELYISSFERHYSLLGT
 E. coli K1         KVLFIPTFPNVFSLKKVIWFYNVYNYLVLNSKAKDAYFMSYAQHYAIFVY
 N. meningitidis B  YLFELPRSPNNITPKKLLYIYRSYKKILNIIQPAHLYMLSFTGHYSYLIS
                     .: :*  *    :. *: : : . *: ::   :. . *: *:  **: :

                                 152EE153 T155
 M. haemolytica A2  LAKNMGFKVNLVEEGTGTYKYSSMQEACKKLDDSMNYQEKKVYKKISKSF
 E. coli K1         LFKKNNIRCSLIEEGTGTYKTEK---------ENPV-VNINFYSE-----
 N. meningitidis B  IAKKKNITTHLIDEGTGTYAPLL---------ESFSYHPTKLERY----L
                    : *: .:   *::******               :.       :.

 M. haemolytica A2  IYKNIRSSLKPFDSFDHIYVAFPEKVKNVFKCNKISFF-SIYESRLENEH
 E. coli K1         IINSIILFHYPDLKFENVYGTYPILLKKKFNAQKFVEF-KGAPSVKSSTR
 N. meningitidis B  IGNNLNI-KGYIDHFDILHVPFPEYAKKIFNAKKYNRFFAHAGGISINNN
                    * :.:           *: :: .:*   *: *:.:*   *      .   . .

                                         R259
 M. haemolytica A2  VSEFIRNNKCSKKNIIFCAQRYPIPEREYISTILDILYKYAKEYKTKVFI
 E. coli K1         IDNVIHKYSITRDDIIYANQKYLIEHTLFADSLISILLRIDKPDNARIFI
 N. meningitidis B  IANLQKKYQISKNDYIFVSQRYPISDDLYYKSIVEILNSISLQIKGKIFI
                    : :. :: . :::: *:   *:* *  .  : .:::.**         : ::**

                   K289 H291 K293                          E323
 M. haemolytica_A2  KLHPKERIET--ID-VYKEISKDK-QGLIIMENISFPAEDFISQLKPRKV
 E. coli K1         KPHPKEPKKN--INAIQKAIKKAKCRDIILITEPDFLIEPVIKKAKIKHL
 N. meningitidis B  KLHPKEMGNNYVMS-LFLNMVEIN-PRLVVINEPPFLIEPLIYLTNPKGI
                    * ****  :.  :.  :   : : :   :::: :  *  *  .*    : : :

                      339STS341                                  H382
 M. haemolytica A2  LSIASTSLVYTTLISKDIKAISIYPLFRKEVLKKIEYKEEYFKDIESHYS
 E. coli_K1         IGLTSSSLVYAPLVSKRCQSYSIAPLMIKLCDN--DKSQKGINTLRLHFD
 N. meningitidis B  IGLASSSLIYTPLLSPSTQCLSIGELIINLIQK--YSMVENTEMIQEHLE
                    :.::*:**:*:.*:*   :. **  *: :   :      :  : :. * .

 M. haemolytica A2  LLSKFDGIRILNNTNE----------------------------------
 E. coli K1         ILKNFDNVKILSDDITS---PSL---------------------------
 N. meningitidis B  IIKKFNFINILNDLNGVISNPLFKTEETFETLLKSAEFAYKSKNYFQAIF
                    ::.:*: :.**.:

 M. haemolytica A2  --------------------------------------------------
 E. coli K1         --------------------------------------------------
 N. meningitidis B  YWQLASKNNITLLGHKALWYYNALYNVKQIYKMEYSDIFYIDNISVDFHS

 M. haemolytica A2  -----------------------I
 E. coli K1         ----------------HDKRIFLGE
 N. meningitidis B  KDKLTWEKIKHYYYSADNRIGRDR
```

Supplementary Figure S4 | Sequence conservation of bacterial α-2,8 polysialyltransferases. Sequence alignment of PSTs from *Manheimia haemolytica A2, Escherichia coli K1*, and *Neisseria meningitidis group B*. Residues essential for catalysis are indicated, coloured in red or blue, and are shaded in grey. Residues with a potential role in catalysis are coloured in red or blue. Alignment was made using T-Coffee [1].

Supplementary Figure S5

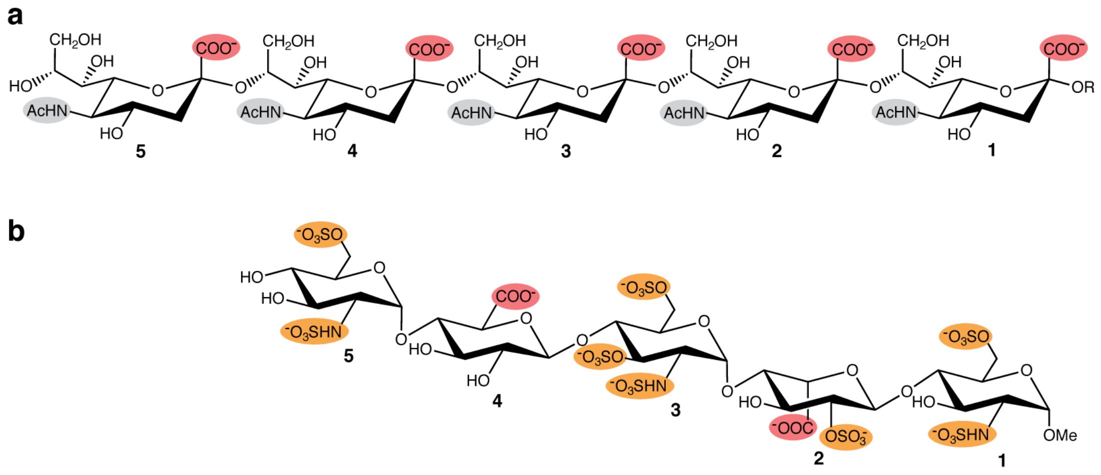

Supplementary Figure S5 | Structural comparison of polysialic acid and fondaparinux. (a) Chemical structure of the D-Neu5Ac pentamer α-(2,8)-Sia_5 (R = H for the plain pentamer, or R = $(Sia)_n$-$(Kdo)_{2\text{-}8}$-lyso-phosphatidyl glycerol for the PST acceptor substrate). (b) Chemical structure of the pentasaccharide heparin-mimetic fondaparinux with the monosaccharide sequence D-GlcNS6S-α-(1,4)-D-GlcA-β-(1,4)-D-GlcNS3,6S-α-(1,4)-L-IdoA2S-α-(1,4)-D-GlcNS6S-OMe. (GlcNS6S is 2-deoxy-6-O-sulfo-2-(sulfoamino)-α-D-glucopyranoside, GlcA is β-D-glucopyranuronoside, GlcNS3,6S is 2-deoxy-3,6-di-O-sulfo-2-(sulfoamino)-α-D-glucopyranoside, IdoA2S is 2-O-sulfo-α-L-idopyranuronoside, and GlcNS6S-OMe is methyl-O-2-deoxy-6-O-sulfo-2-(sulfoamino)-α-D-glucopyranoside.) (a, b) Numbers below the monosaccharides indicate the orientation of the pentasaccharide with respect to the reducing end residue. To illustrate the charge distribution of the two molecules, carboxyl groups are highlighted in red, sulfo and sulfoamino groups are highlighted in orange, and N-acetyl groups are highlighted in grey.

Supplementary Figure S6

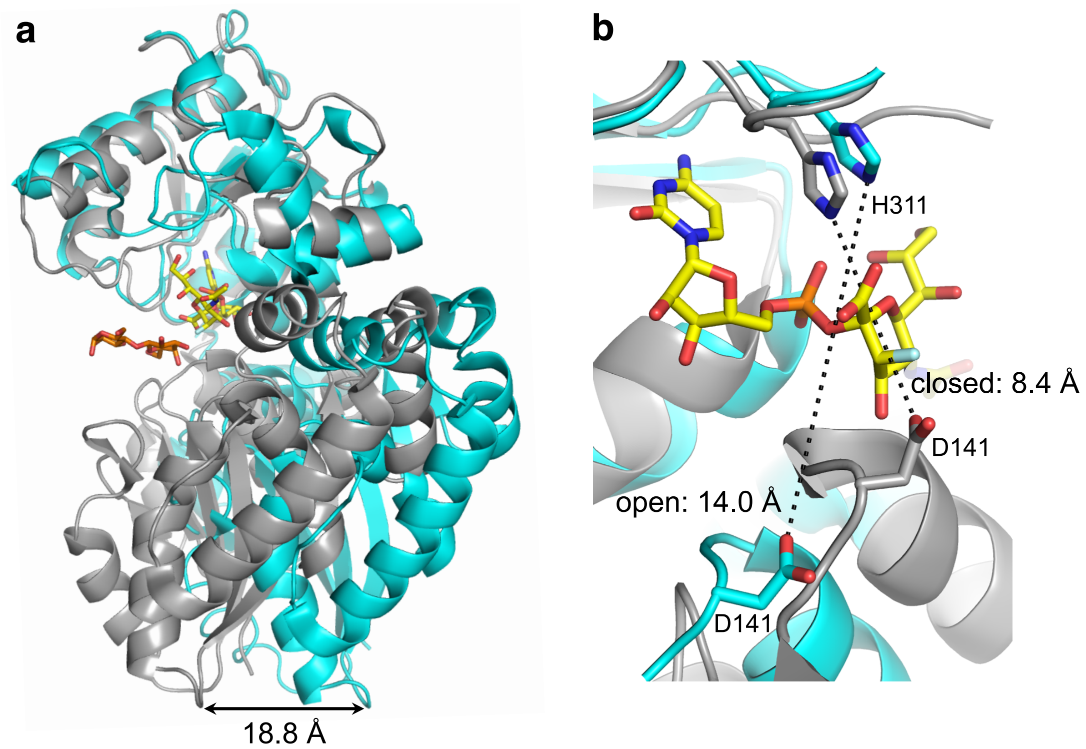

Supplementary Figure S6 | Conformational changes in mono-sialyltransferase PmST1 upon substrate binding. (a) The open conformation of PmST1 is shown in cyan (PDB: 3s44), the closed conformation is shown in gray (PDB: 2ihz), bound CMP-3F(*e*)Neu5Ac is coloured in yellow and bound lactose is coloured in orange. The two structures were superimposed based on their C-terminal domain, to show the large conformational change upon substrate binding. (b) Close-up view of the active site with catalytic residues D141 and H311 shown as sticks. The distance between the catalytic residues is shown for both structures.

Supplementary Figure S7

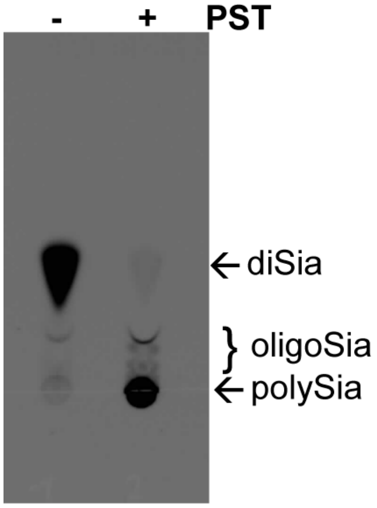

Supplementary Figure S7 | *In vitro* polysialylation activity of *Mh*PST. The activity of purified *Mh*PST was tested in an *in vitro* reaction using a soluble BODIPY-diSiaLac acceptor and CMP-Neu5Ac donor. Product formation was analysed by TLC, where polysialylation results in suppressed migration of the acceptor. TLC plates were illuminated under UV light to visualize acceptor substrate conversion.

Supplementary References

1 Di Tommaso, P. *et al.* T-Coffee: a web server for the multiple sequence alignment of protein and RNA sequences using structural information and homology extension. *Nucleic Acids Res* **39**, W13-17, doi:10.1093/nar/gkr245 (2011).
